# Phenotypic plasticity in fish cell lines: conceptual links to aquatic epigenetics

**DOI:** 10.3389/freae.2026.1867376

**Published:** 2026-06-24

**Authors:** Daisuke Ikeda

**Affiliations:** School of Marine Biosciences, Kitasato University, Sagamihara, Kanagawa, Japan

**Keywords:** cellular senescence, cultivated meat, cultivated seafood, epigenetic plasticity, fish cell line, long-term culture, phenotypic plasticity, spontaneous immortalization

## Abstract

Fish cell lines have long supported aquatic research, yet their emerging role as seed cells for cultured seafood requires a deeper understanding of their phenotypic stability. Although these cells often demonstrate prolonged proliferation and relative resistance to culture-associated senescence, they can also exhibit marked culture-dependent variability. In this review, I examine a central feature of fish cell culture: the coexistence of durable growth and flexible cell states. By focusing on spontaneous immortalization, passage-dependent changes, and differentiation-related plasticity, particularly in muscle and adipose lineages, I consider how these phenomena may be interpreted through the lens of epigenetic plasticity. Direct epigenomic evidence remains limited, and this review therefore presents a hypothesis-generating conceptual framework rather than an established mechanistic model. Nevertheless, studies involving DNA demethylation, incomplete senescence programs, possible differences in senescence-related genomic context, environmental epigenetics, and nuclear organization provide conceptual clues that reversible regulatory processes above the level of DNA sequence may shape fish cell-state behavior. I further discuss how this perspective is relevant to aquatic biotechnology and cultured seafood, where reproducibility, lineage control, quality control, and process standardization depend on monitoring cell-state behavior while distinguishing reversible regulatory changes from genetic alterations acquired during long-term culture. Clarifying the regulatory processes associated with phenotypic plasticity in fish cell lines may therefore provide a useful foundation for future epigenomic studies and for the development of biologically reliable fish cell platforms.

## 1 Introduction

Epigenetic regulation has become an important framework for understanding how aquatic organisms respond to developmental and environmental cues without changes in DNA sequence (Gavery and Roberts, 2017; Lu et al., 2026). In aquatic biology, DNA methylation, histone modification, chromatin remodeling, and non-coding RNA are increasingly discussed in relation to growth, reproduction, stress responses, disease resistance, and other traits of practical relevance (Gavery and Roberts, 2017; Lu et al., 2026).

Fish-derived cell lines have long been used in virology, toxicology, endocrinology, and related fields (Lakra et al., 2011; Kumar et al., 2024), and more recently have attracted attention as seed cells for cultured seafood (Rubio et al., 2019; Xue et al., 2024). Compared with many commonly used mammalian systems, fish cells are often maintained at relatively low temperatures and may possess physiological properties advantageous for *in vitro* cultivation (Rubio et al., 2019). Recent studies have further expanded the repertoire of fish muscle-related cell lines, including continuous or spontaneously immortalized lines that retain myogenic or adipogenic characteristics over extended culture (Ikeda et al., 2024; Xue et al., 2024; Kishino et al., 2025; Li et al., 2025).

Nevertheless, fish cell lines remain only loosely integrated into current discussions of aquatic epigenetics. This is notable because long-term culture often involves substantial changes in proliferative behavior, differentiation-related phenotypes, and responses to culture conditions. In Japanese eel primary muscle cultures, myoblast-rich populations declined with passaging and were progressively replaced by fibroblast-like cells, whereas early single-cell cloning enabled the establishment of a stable myoblast line, JEM1129 (Ikeda et al., 2024). In rainbow trout and Japanese eel, continuous or spontaneously immortalized cell lines retained differentiation-related properties over extended culture while still exhibiting heterogeneity and culture-dependent behavior (Kishino et al., 2025; Li et al., 2025). In EPC cells, a well-established epithelioma papulosum cyprini-derived fish cell line, treatment with the DNA demethylating agent 5-aza-2′-deoxycytidine induced senescence-like phenotypes and senescence-associated secretory features, suggesting that at least some aspects of fish cell behavior may be influenced by regulation above the level of DNA sequence (Futami et al., 2019).

In this review, I discuss spontaneous immortalization, plasticity during prolonged culture, and changes in differentiation-related potential in fish cell lines. Rather than proposing a single mechanism to explain these behaviors, I consider whether they can be understood within a broader, hypothesis-generating framework for epigenetic plasticity. This framework may offer a useful starting point for future epigenomic studies of fish cell systems. The conceptual framework linking teleost growth characteristics, culture-dependent cell-state changes, candidate processes to be tested, and applied relevance to cultured seafood is summarized in Figure 1.

**FIGURE 1:**
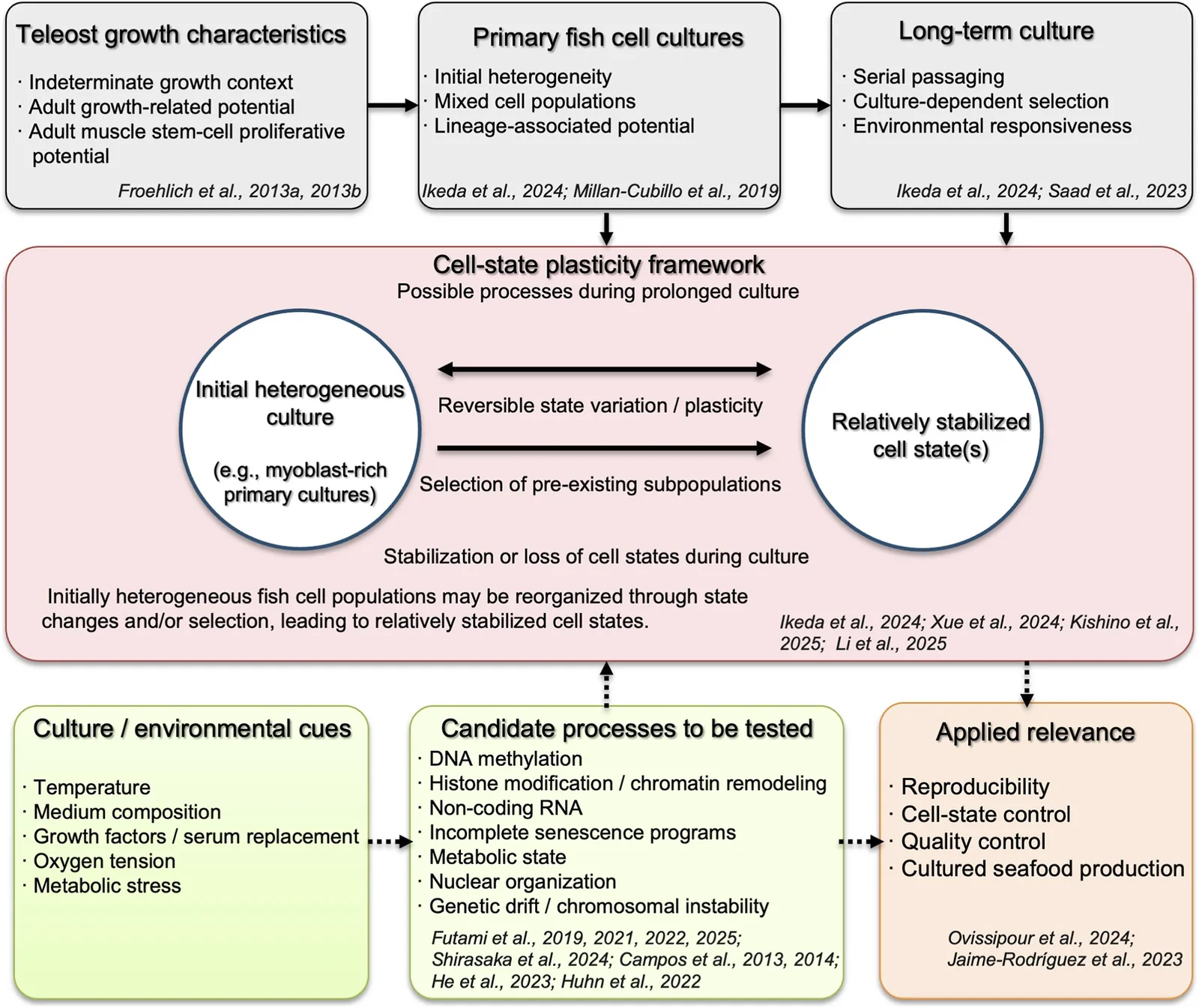
Conceptual framework linking long-term proliferation, phenotypic plasticity, and candidate regulatory processes in fish cell lines. Fish cell cultures often begin as heterogeneous populations and may be reshaped during prolonged *in vitro* culture by serial passaging, culture-dependent selection, and environmental responsiveness. The central panel summarizes a working model in which initially heterogeneous fish cell populations may be reorganized through state variation/plasticity and/or selection of pre-existing subpopulations, leading to stabilization or loss of particular cell states and the emergence of relatively stabilized culture outcomes. Candidate processes to be tested include DNA methylation, histone modification and chromatin remodeling, non-coding RNA, incomplete senescence programs, metabolic state, nuclear organization, genetic drift, and chromosomal instability. These processes may contribute to cell-state stability or plasticity, or may provide alternative explanations for phenotypic changes observed during long-term culture. This framework is relevant to cultured seafood because reproducibility, cell-state control, quality control, and process standardization depend on understanding and managing cell-state behavior during long-term culture. Solid arrows indicate descriptive relationships in the culture process or possible processes summarized within the framework, whereas dashed arrows indicate hypothetical or indirect relationships that remain to be tested.

## 2 Unique biological features of fish cell lines

Fish cell lines often exhibit characteristics that set them apart from many commonly used mammalian culture systems. Long-term propagation of fish-derived cells from normal tissues has been reported for decades, and fish cell lines have been widely used in aquatic biology and biotechnology (Lakra et al., 2011; Kumar et al., 2024). Early examples include fibroblast-like lines derived from goldfish and medaka fins, such as RBCF-1 and OL-17/OL-32, which demonstrated prolonged *in vitro* growth without deliberate transformation (Shima et al., 1980; Komura et al., 1988). Similarly, cells from the short-lived killifish Nothobranchius furzeri were reported to lack clear replicative senescence *in vitro* (Graf et al., 2013). These reports make it difficult to view extended proliferative capacity as an exceptional feature limited to a few recently established cultured seafood-related fish cell lines (Shima et al., 1980; Komura et al., 1988; Lakra et al., 2011; Kumar et al., 2024). It appears instead to be a more general tendency in fish cell culture, even if its mechanistic basis differs among systems.

To avoid conceptual ambiguity, several related terms should be distinguished. In this review, long-term proliferation or prolonged propagation is used descriptively to refer to sustained growth over repeated passages. Continuous culture refers to maintenance of a cell population through serial subculture under defined conditions. Spontaneous immortalization is used more specifically to indicate acquisition of an apparently indefinite proliferative capacity without deliberate genetic transformation. These terms are related but not interchangeable; extended propagation alone should not be regarded as direct evidence of immortalization.

One possible biological context for this pattern is the life-history strategy of many teleosts. Unlike mammals, many fish continue growing throughout life and retain substantial growth potential in adult tissues. This indeterminate growth paradigm has been discussed in relation to the proliferative and self-renewal capacity of adult muscle stem cells and the continued recruitment of new muscle fibers in adult fish (Froehlich et al., 2013a). A related *in vitro* study using giant danio further supported the view that growth-related programs can remain active in adult fish muscle (Froehlich et al., 2013b). These studies alone do not explain spontaneous immortalization. They do, however, make it easier to understand why proliferative restraint and lineage maintenance might not operate in fish cells exactly as they do in mammalian systems.

Fish cells may also be attractive for *in vitro* production systems because they exhibit tolerance to hypoxia, substantial buffering capacity, and growth at relatively low temperatures (Rubio et al., 2019). Recent studies relevant to cultured seafood reinforce this picture. Examples include a spontaneously immortalized muscle stem cell line with prolonged myogenic stemness and adipogenic trans-differentiation capacity in brown-marbled grouper (Xue et al., 2024), continuous muscle- and adipose-derived stem cell lines in rainbow trout (Li et al., 2025), a stable myoblast line recovered by single-cell cloning in Japanese eel (Ikeda et al., 2024), and spontaneously immortalized eel muscle-derived preadipocyte lines with sustained lipid accumulation and adipogenic differentiation potential (Kishino et al., 2025). Together, these examples indicate that prolonged propagation and lineage-related functionality can coexist in fish-derived cultured cells, at least under some culture conditions.

At the same time, prolonged proliferative capacity does not necessarily imply phenotypic stability. Changes in morphology, lineage-marker expression, differentiation behavior, or subpopulation structure may accompany extensive passaging. In Japanese eel primary muscle cultures, myoblast-rich populations declined during passaging and were progressively replaced by fibroblast-like cells, after which a stable myoblast line was recovered by early single-cell cloning (Ikeda et al., 2024). Thus, one of the most distinctive aspects of fish cell lines may be the coexistence of relative ease of long-term propagation and marked variability or plasticity of cell state.

## 3 Phenotypic plasticity observed during long-term culture

Phenotypic plasticity in fish cell culture is expressed not only as differences between cell lines, but also as dynamic changes within a given culture over time. In this review, phenotypic plasticity is used as an umbrella term for culture-associated changes in observable cellular properties, including morphology, lineage-marker expression, differentiation competence, and proliferative behavior. Importantly, this term does not necessarily imply direct conversion of one cell type into another. Such changes may arise through at least three non-exclusive processes: true cell-state transition within individual cells, selective expansion of pre-existing subpopulations, and culture adaptation during serial passaging. Distinguishing among these processes is essential when interpreting passage-dependent changes in heterogeneous fish cell cultures. In this context, prolonged culture may be accompanied by shifts in morphology, lineage-marker expression, proliferative behavior, and differentiation capacity, rather than the simple maintenance of a fixed identity. Fish primary cultures often begin as heterogeneous populations and may then undergo selective expansion, subpopulation drift, or possible shifts in cellular state, depending on the tissue of origin and culture conditions. In addition, primary myogenic cultures in fish can provide a relatively extended *in vitro* time window in which progression from proliferation to differentiation can be followed. In primary cultures of gilthead sea bream myocytes, for example, mononucleated proliferative cells predominated early, whereas multinucleated myotubes emerged later, accompanied by marked shifts in myogenin expression and broader proteomic profiles (Millan-Cubillo et al., 2019). Although this study primarily describes staged myogenesis rather than cell-state instability, it illustrates that fish myogenic progression *in vitro* can be resolved over a comparatively extended developmental timescale.

Japanese eel muscle-derived primary cultures provide a clear example. Myoblast-rich populations were present early after isolation, but continued passaging led to a decline in myoblastic characteristics and an increase in fibroblast-like cells. Transcriptomic and RT-qPCR analyses showed that myogenic markers, including *pax7a* and *myoD*, decreased with passage number, indicating that the phenotypic shift was not merely morphological (Ikeda et al., 2024). In the original study, fibroblast contamination and subsequent overgrowth were discussed as one plausible explanation, because even a small number of fibroblasts may outcompete myoblast-rich populations during culture. At the same time, studies in porcine satellite cells have suggested that even purified satellite cells can lose stem-like or myogenic characteristics during passaging (Ding et al., 2017; Zhu et al., 2022). Although these studies were not conducted in fish, they raise the possibility that passage-dependent loss of myogenic traits in eel cultures may not be explained solely by fibroblast overgrowth. Early single-cell cloning enabled the recovery of a stable myoblast line, JEM1129, indicating that long-term fish cell culture may involve both selective expansion of pre-existing cell populations and culture-dependent stabilization of particular cell states, although their relative contributions remain unresolved.

Plasticity is also evident in proliferative fish cell lines. In rainbow trout, RTMS and RTADS retained myogenic or adipogenic potential over extended passage, but efficient differentiation still depended on medium optimization (Li et al., 2025). In brown-marbled grouper, prolonged stemness and adipogenic trans-differentiation coexisted in a single muscle-derived line (Xue et al., 2024). In Japanese eel preadipocyte lines, sustained proliferation was accompanied by line-dependent differences in zeta potential, PPARG responsiveness, oleic acid sensitivity, and adipogenic behavior (Kishino et al., 2025). In some systems, these processes may also include a recognizable crisis-like phase before long-term proliferative stabilization, as reported for a continuous Atlantic mackerel muscle cell line with myogenic- and adipogenic-like phenotypes (Saad et al., 2023).

Overall, long-term fish cell culture often yields systems in which proliferative continuity coexists with substantial cell-state flexibility. This raises basic biological questions about how lineage identity is stabilized or destabilized *in vitro*, as well as practical questions about reproducibility, clonal consistency, and process predictability in aquatic biotechnology and cultured seafood production.

## 4 Conceptual links to epigenetic plasticity

The phenotypic behaviors described above are difficult to interpret solely as the consequence of fixed genetic change. In many fish cell systems, prolonged proliferation coexists with gradual, conditional, or lineage-dependent changes in morphology, lineage-associated traits, and differentiation competence. Such features are broadly consistent with the idea that at least part of fish cell behavior *in vitro* reflects plastic regulation of cell state rather than irreversible lineage commitment alone.

Direct epigenomic evidence remains limited in most fish cell lines, and it would be premature to claim that the observed plasticity is epigenetically driven in any single mechanistic sense. Nevertheless, studies using EPC cells provide important conceptual clues. At present, EPC remains one of the most mechanistically informative fish cell systems for discussing senescence-related regulation, although whether the same regulatory logic applies broadly across other fish cell lineages remains unresolved. In this system, pharmacological DNA demethylation by 5-aza-2′-deoxycytidine induced senescence-like phenotypes and senescence-associated secretory features (Futami et al., 2019), suggesting that methylation-dependent regulation may contribute to suppression of senescence-related programs in at least some fish cells.

Comparative observations across multiple fish cell cultures provide a broader context. Many long-standing fish cell lines showed little or no senescence-associated β-galactosidase staining. In contrast, in some newly developing cultures, SA-β-gal-positive cells were present early and were later replaced by proliferative SA-β-gal-negative populations (Vo et al., 2015). These findings suggest that senescence-associated states in fish cell culture may be incomplete, heterogeneous, or transient rather than uniformly stabilized.

Studies using EPC cells provide further mechanistic clues. Early, but not full, senescence was induced by increased H-Ras expression (Futami et al., 2021), and the absence of a *p16* ^*INK4a*^ */ARF* locus in the fish genome was proposed as one possible determinant of this phenotype (Futami et al., 2022). Mitochondrial dysfunction induced several senescence-associated features, including SA-β-gal activity and proinflammatory SASP-related signals, but not irreversible growth arrest unless combined with constitutively active H-Ras (Shirasaka et al., 2024). In addition, heterologous expression of human PML-IV induced premature senescence-like phenotypes together with PML nuclear body-like structures and proinflammatory SASP-associated changes, implicating nuclear organization and state-dependent transcriptional regulation as additional factors potentially relevant to fish cell senescence resistance (Futami et al., 2025). These EPC-based studies are important because they provide rare mechanistic insight into senescence-related regulation in fish cells. However, they should not be interpreted as evidence that the same regulatory mechanisms operate broadly in all fish cell lines. Whether similar mechanisms apply to muscle-derived, adipose-derived, or cultured seafood-related fish cell lines remains to be directly tested.

Studies of environmental epigenetics in teleost muscle provide a supportive, though indirect, context for this framework. In Senegalese sole larvae, altered rearing temperature affected DNA methylation of the myogenin promoter together with myogenin expression and muscle cellularity (Campos et al., 2013). In the same species, embryonic temperature reshaped the developmental miRNA transcriptome, including several miRNAs implicated in myogenesis and growth regulation (Campos et al., 2014). Likewise, in zebrafish, embryonic temperature affected adult muscle fiber recruitment, and genome-wide changes in mRNA and miRNA expression were identified during the transition from hyperplastic to hypertrophic growth phenotypes (Johnston et al., 2009). Although these studies were not performed in fish cell lines, they support the broader view that cellular identity and developmental state in teleost muscle remain responsive to regulatory reorganization above the level of DNA sequence.

Taken together, these studies do not establish a unified epigenetic mechanism for the plasticity of fish cell lines. Rather, they support a conceptual model in which extended proliferation, incomplete senescence, lineage drift, conditional differentiation, and heterogeneity among related lines may be shaped, at least in part, by reversible regulatory processes operating above the level of DNA sequence.

## 5 Discussion

The studies discussed above suggest that fish cell lines represent unusual biological systems: They often combine prolonged proliferative capacity with marked variability in morphology, differentiation-related properties, and responses to culture conditions. This combination makes them valuable experimental tools, but it also complicates interpretation, because long-term propagation does not necessarily imply stable cellular identity. One important issue is whether the behaviors observed during long-term culture should be understood primarily as consequences of intrinsic features of fish biology, such as indeterminate growth and sustained myogenic potential in adult tissues, or as primarily reflecting selection imposed by *in vitro* culture itself. The most plausible interpretation is that fish cell line behavior emerges from an interaction between these two factors, in which biologically permissive starting states are progressively shaped, stabilized, or lost under culture conditions.

A central unresolved question concerns the interpretation of phenotypic change within mixed or passaged cultures. In some systems, such changes may reflect selective expansion of pre-existing subpopulations rather than direct conversion of one cell type into another. In others, conditional preservation or reactivation of lineage-associated traits suggests that transitions in cell state may remain possible even after prolonged expansion. At present, available data support the plausibility of both processes, but their relative contributions remain unresolved. In Japanese eel muscle cultures, passage-dependent loss of myogenic characteristics and recovery of a stable myoblast line by early single-cell cloning support the idea that culture history can influence which cellular state becomes dominant (Ikeda et al., 2024).

Another important unresolved issue is the distinction between epigenetic plasticity and genetic changes accumulated during prolonged culture. Phenotypic drift in continuous cell cultures may reflect not only reversible regulatory changes but also genetic drift, chromosomal instability, or clonal selection. Cell-line studies more broadly indicate that chromosomal instability can generate genetic heterogeneity and unstable cellular properties during culture (He et al., 2023), and multi-omics analysis of mammalian CHO bioproduction lineages has shown that culture conditions and selection criteria can shape heritable genomic and phenotypic traits during cell-line adaptation (Huhn et al., 2022). Although these studies were not performed in fish cell lines, they highlight the need to evaluate genome-level changes in parallel with transcriptomic and epigenomic analyses. Future studies of fish cell lines should therefore incorporate whole-genome sequencing, copy-number analysis, and chromosome-level assessment where feasible, together with single-cell RNA sequencing to resolve cellular heterogeneity, subpopulation shifts, and possible cell-state transitions. Such integrated analyses will be necessary to distinguish reversible cell-state regulation from genetic alterations acquired during long-term culture.

In light of these limitations, the epigenetic framework proposed here should be interpreted with caution, even though EPC-based studies provide useful mechanistic clues. Pharmacological DNA demethylation with 5-aza-2′-deoxycytidine induced senescence-like phenotypes and senescence-associated secretory features in EPC cells (Futami et al., 2019), and subsequent studies suggested that fish cell senescence resistance may involve incomplete engagement of canonical senescence pathways, altered maturation of senescence-related states, and possibly nuclear organizational factors (Futami et al., 2021; 2022; 2025; Shirasaka et al., 2024). However, direct epigenomic analyses remain scarce in most fish cell systems, and it is still too early to define a unified epigenetic mechanism for spontaneous immortalization, lineage drift, or conditional differentiation. The framework proposed here should therefore be regarded as hypothesis-generating and provisional. Its value lies in organizing currently scattered observations and identifying testable questions, rather than in establishing a unified epigenetic mechanism for fish cell line plasticity.

These unresolved issues have immediate implications for aquatic biotechnology and cultured seafood. Prolonged proliferation is valuable because it can provide a stable supply of cells for repeated experimentation and potential scale-up. However, plasticity in cell state introduces uncertainty because morphological drift, altered differentiation competence, and shifts in subpopulation structure may reduce reproducibility and make culture outcomes less predictable. In cultivated seafood, general production workflows include cell selection, media preparation, proliferation and differentiation, harvesting, and downstream food processing, with quality-control issues arising at multiple stages (Ovissipour et al., 2024). In cultivated meat systems more broadly, passage-dependent genetic drift, altered functional properties, and loss of stemness have been identified as challenges during the expansion of adherent stem-like cells (Jaime-Rodríguez et al., 2023). In fish systems specifically, passage-dependent shifts in lineage-associated characteristics and phenotypic divergence among cultured lines have been observed in Japanese eel cultures, indicating that prolonged culture may alter not only growth properties but also the biological meaning of the cells being expanded (Ikeda et al., 2024; Kishino et al., 2025). Future studies should therefore monitor a defined set of cell-state and process indicators, such as passage number, population doubling time, cell morphology, differentiation efficiency, expression of myogenic and adipogenic markers, genomic stability, DNA methylation profiles, and single-cell state composition. Tracking these parameters would help define acceptable passage windows and identify culture conditions that preserve reproducible proliferation and differentiation.

Several research needs stand out clearly. First, integrated genomic, transcriptomic, and epigenomic profiling is needed across fish cell lines with different proliferative behavior, lineage stability, and differentiation competence. Second, lineage-tracing or single-cell approaches, including single-cell RNA sequencing, will be important for distinguishing subpopulation selection from true state transition within heterogeneous cultures. Third, more standardized benchmarking of cell-state drift during passage is needed. Finally, mechanistic work should be expanded beyond a small number of model systems, especially beyond EPC cells. A major limitation of the current literature is that senescence-related mechanisms have been investigated in detail mainly in EPC cells, whereas comparable analyses remain scarce in muscle-derived, adipose-derived, and cultured seafood-related fish cell lines. Progress in this area will also benefit from broader access to diverse fish cell models. In this context, ongoing efforts in our laboratory to develop cell lines from more than 20 fish species may provide a useful comparative platform for future studies of cell-state stability and epigenetic regulation across teleosts.

Future developments will likely depend on linking descriptive culture phenotypes to higher-resolution molecular analysis. Fish cell lines should not only be classified by simple descriptors such as proliferative capacity or lineage-associated phenotype, but also characterized in terms of how stable those states are over time and under changing environmental conditions. Environmental variables such as temperature have been shown in teleost models to affect DNA methylation of myogenic regulators, developmental miRNA landscapes, muscle fiber recruitment, and growth-related phenotypes (Johnston et al., 2009; Campos et al., 2013; 2014). Future work will therefore benefit from integrating cell biology with genomics, transcriptomics, epigenomics, single-cell analysis, and bioprocess engineering.

In summary, fish cell lines represent unusual model systems in which long-term proliferation, incomplete senescence, and phenotypic plasticity coexist. Although the mechanistic contribution of epigenetic regulation remains incompletely resolved, the available evidence supports the view that reversible regulatory processes may contribute to fish cell behavior *in vitro*, alongside possible genetic changes acquired during prolonged culture. This perspective is relevant not only to basic fish cell biology but also to the development of standardized, biologically reliable fish cell platforms for aquatic biotechnology and cultured seafood.
